# The impact of super-spreader cities, highways, and intensive care availability in the early stages of the COVID-19 epidemic in Brazil

**DOI:** 10.1038/s41598-021-92263-3

**Published:** 2021-06-21

**Authors:** Miguel A. L. Nicolelis, Rafael L. G. Raimundo, Pedro S. Peixoto, Cecilia S. Andreazzi

**Affiliations:** 1 Department of Neurobiology, Duke University Medical Center, Box 103905, Durham, NC 27710 USA; 2 Department of Biomedical Engineering, Duke University, Durham, NC USA; 3 Department of Neurology, Duke University, Durham, NC USA; 4 Department of Neurosurgery, Duke University, Durham, NC USA; 5 Department of Psychology and Neuroscience, Duke University, Durham, NC USA; 6 Edmond and Lily Safra International Institute of Neurosciences, Natal, Brazil; 7 Department of Engineering and Environment and Postgraduate Program in Ecology and Environmental Monitoring (PPGEMA), Center for Applied Sciences and Education, Federal University of Paraíba-Campus IV, Rio Tinto, Paraíba, Brazil; 8 Department of Applied Mathematics, Institute of Mathematics and Statistics, University of São Paulo, São Paulo, Brazil; 9 Laboratory of Biology and Parasitology of Wild Reservoir Mammals, IOC, Oswaldo Cruz Foundation, Rio de Janeiro, Brazil

**Keywords:** Diseases, Infectious diseases

## Abstract

Although international airports served as main entry points for SARS-CoV-2, the factors driving the uneven geographic spread of COVID-19 cases and deaths in Brazil remain mostly unknown. Here we show that three major factors influenced the early macro-geographical dynamics of COVID-19 in Brazil. Mathematical modeling revealed that the “super-spreading city” of São Paulo initially accounted for more than 85% of the case spread in the entire country. By adding only 16 other spreading cities, we accounted for 98–99% of the cases reported during the first 3 months of the pandemic in Brazil. Moreover, 26 federal highways accounted for about 30% of SARS-CoV-2’s case spread. As cases increased in the Brazilian interior, the distribution of COVID-19 deaths began to correlate with the allocation of the country’s intensive care units (ICUs), which is heavily weighted towards state capitals. Thus, severely ill patients living in the countryside had to be transported to state capitals to access ICU beds, creating a “boomerang effect” that contributed to skew the distribution of COVID-19 deaths. Therefore, if (i) a lockdown had been imposed earlier on in spreader-capitals, (ii) mandatory road traffic restrictions had been enforced, and (iii) a more equitable geographic distribution of ICU beds existed, the impact of COVID-19 in Brazil would be significantly lower.

## Introduction

Barely 6 months after its first report of a COVID-19 case, on February 26th, 2020, Brazil recorded the staggering tally of more than 4,000,000 cases and 125,000 deaths^1^ as a consequence of the rampant SARS-CoV-2 epidemic that raged through the entire country. Those numbers ensure that, by September 16th, Brazil was the third most affected country globally, right behind the United States and India in terms of both accumulated COVID-19 cases, and second only to the US in terms of deaths^1^. By early March, it became clear that the country’s international airports, located mainly in large state capital cities on the Brazilian Atlantic coast (with only three main exceptions: Brasilia, Belo Horizonte, and Manaus) had been the main entry points of SARS-CoV-2 into the country^2^. However, even though the main coronavirus genotypes arriving and spreading through the country were rapidly identified^3^, the routes taken by SARS-CoV-2 to reach the entire Brazilian territory remained mysterious until now. In addition, the heavily skewed and heterogeneous spatial distribution of COVID-19 cases throughout the country’s five official regions [North (NO), Northeast (NE), Central-West (CO), Southeast (SE), and South (S)], even after 6 months of an out of control epidemic, as well as the discrepancy between cases and death distributions caught our attention.

From the biogeographical standpoint, one can analyze the new coronavirus spread in Brazil as the anthropogenic mass dispersal of a zoonotic agent expanding its geographical range. Such a process typically involves multiple sources and entry points that follow several dispersal pathways, through which the biological invasion propagates over space^4^. Understanding the geographical spread of directly transmitted infectious diseases into uninfected regions requires one to focus on human and pathogen population traits, instead of environmental factors, since the former are the primary drivers of the biological invasion^5^. Indeed, environmental factors, such as climate or temperature variations, are poorly related to the large-scale geographical spread of the new coronavirus, since SARS-CoV-2 seems to expand its geographical range mostly by the flow of people in transportation networks^6,7^. Therefore, to unravel the pathways that shaped the geographical distribution of COVID-19 cases and deaths in Brazil, we examined human-mediated processes that turned particular capital cities into SARS-CoV-2 “*superspreaders”* at the geographical scale, while leading other towns to act only as entry points of the pandemic and limiting their contribution to the local spreading of the infection. As such, the present work aims at enlightening the fundamental processes that accounted for the macro-geographical patterns of COVID-19 cases and deaths arising from the rapid spread of the new coronavirus over large continental areas in Brazil during the first stages of the pandemic. Therefore, in this first study, we did not consider local and regional dynamics, which are certainly relevant for the understanding of patterns at coarser spatial scales but are beyond the scope of this study.

Although often considered a key parameter in mathematical models of epidemics^8–11^, population density alone does not necessarily determine which cities will become superspreaders^12^. This happens because cities are embedded within complex transportation networks whose connectivity plays a major role in determining the geographical spread of infected individuals and, hence, in shaping emerging epidemiological patterns at broader scales^13^. The influence of road transportation networks on infectious disease spread is a well-documented phenomenon worldwide (e.g.^14–19^). In Brazil, previous work addressed the spread of SARS-CoV-2 at the state scale (e.g.^20,21^). However, how SARS-Cov-2 rapidly spread over the entire Brazilian federal road network to shape highly uneven patterns of COVID-19 cases and deaths over a complex and heterogeneous country remains an unaddressed question.

While most of the travel network can be halted during a pandemic, essential transportation and travel must be maintained at all costs. For instance, specialized healthcare facilities, such as intensive care units (ICUs), are unevenly distributed in Brazil^22–25^. Therefore, health-related travel is vital for delivering effective patient care. Consequently, in Brazil ICU facilities are frequently shared, not only locally and regionally, but also beyond the state level. Here, we hypothesized that this pattern was likely a key driver of the uneven geographical distribution of COVID-19 deaths in Brazil. The aims of the present study, therefore, were two-fold: (1) to identify the spreading cities and Brazilian federal roads that contributed to the definition of the geographical patterns of COVID-19 cases in Brazil; and (2) to understand how the distribution of ICU beds across the country contributed to the uneven spatial distribution of COVID-19 deaths during the first 6 months of the pandemic in Brazil.

## Results

**Figure 1:**
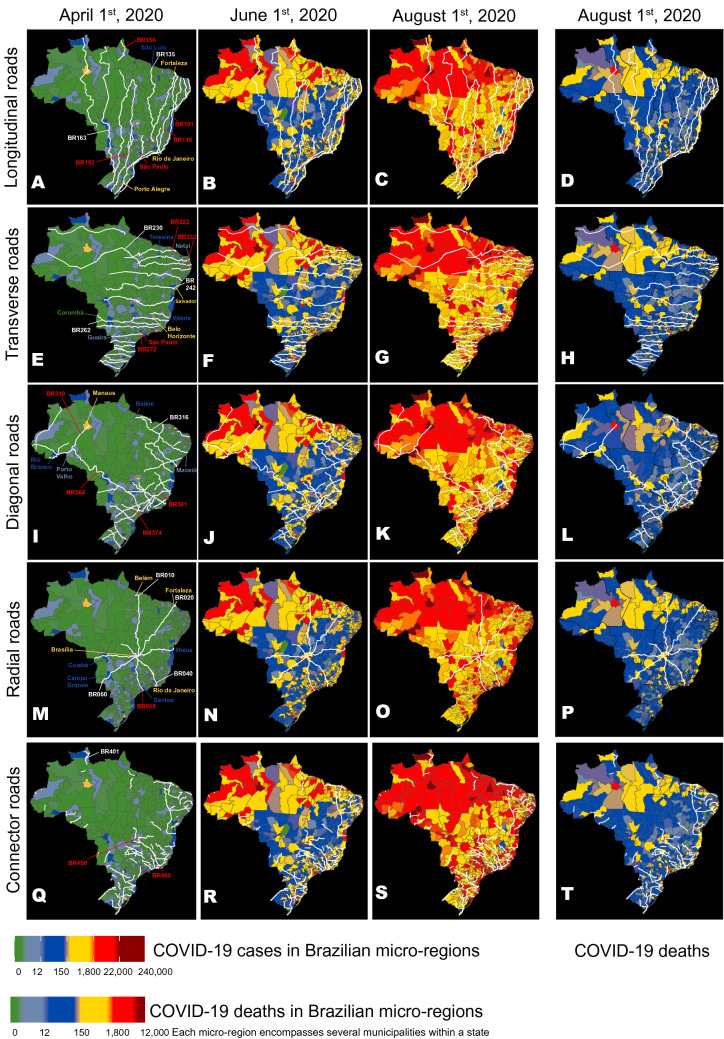
Maps of Brazil were used to represent the routes of the main longitudinal (**A**–**D**), transversal (**E**–**H**), diagonal (**I**–**L**), radial (**M**–**P**), and connector (**Q**–**T**) federal highways, as well as the evolution of the geographic distribution of COVID-19 cases on three dates (April 1st, June 1st, and August 1st), and the distribution of COVID-19 deaths on August 1st (**D**). Overall, 26 highways (see text) from all five road categories contributed to approximately 30% of the COVID-19 case spreading throughout Brazil. The numbers of some of these spreading highways are highlighted in red. Notice how many hotspots (red color) for COVID-19 cases occur in micro-regions containing cities located along major highway routes like BRs 101, 116, 222, 232, 236, 272, 364, 374, 381, 010, 050, 060, 450, and 465. Although the distributions for COVID-19 cases and deaths were correlated, geographic discrepancies between the two distributions can be seen by comparing them on August 1st (**C**,**D**). A color code (see figure bottom) ranks Brazilian micro-regions (each comprising several tows) according to their number of COVID-19 cases and deaths. Maps generated using the R (https://cran.r-project.org/) package brazilmaps 0.1 by R. P. Siqueira (https://github.com/rpradosiqueira/brazilmaps).

Figure 1 compares the distributions of all cases and deaths for all 5570 Brazilian cities from April 1st until August 1st, 2020. A simple visual inspection of such distributions reveals striking spatial patterns in each of them and also a clear dissimilarity among them. For instance, while by August 1st most of the country was reporting a high number of COVID-19 cases, a larger incidence of fatalities was concentrated on the coastal state capitals and medium-sized interior towns (see Fig. 1C,D). To account for such features, we first analyzed the spatial spread of COVID-19 cases and deaths over time through the extensive network of highways that crisscross the whole Brazilian territory, including the North region's vast rain forest. Figure 1A–T illustrates the temporal evolution of the spread of COVID-19 cases over Brazilian micro-regions (each containing several towns) plotted on top of the routes taken by all longitudinal (north–south, Fig. 1A–D), transversal (east–west, Fig. 1E–H), diagonal (Fig. 1I–L), radial (Fig. 1M–P), and connector (Fig. 1Q–T) Brazilian federal highways. Beginning with the early phase of the epidemic (April 1st), one can easily spot the spread of COVID-19 cases across the cities either crossed or located near the routes of two major longitudinal highways (BR 101 and BR 116, Fig. 1A–D) that run from the southern-most state of the country, Rio Grande do Sul (RS), to the north coast states of the NE region. Subsequent snapshots in time (June 1st and August 1st) show COVID-19 cases climbing in cities along other major highways, which became hotspots for the epidemic. Using a multivariate linear model, we observed that a set of 26 federal highways significantly contributed to approximately 30% of the initial COVID-19 spread throughout Brazil (see Supplementary Table 1). In addition to BRs 101 and 116, these included other longitudinal (BRs 153, 156, Fig. 1A–D), transversal (BRs 222, 226, 232, 272, Fig. 1E–H), diagonal (BR 316, 319, 324, 364, 374, 381, Fig. 1I–L), radial (10, 20, 40, 50, 60, Fig. 1M–P), and connector (BR 401, 408, 425, 447, 448, 450, 460, Fig. 1Q–T) federal highways. Similarly, a set of federal highways (BRs 101, 116, 222, 232, 272, 308, 319, 374, 381, 20, 40, 50, 408, 447, 450, and 465) was highly correlated with the distribution of COVID-19 deaths across the whole country (Supplementary Table 2).

**Figure 2:**
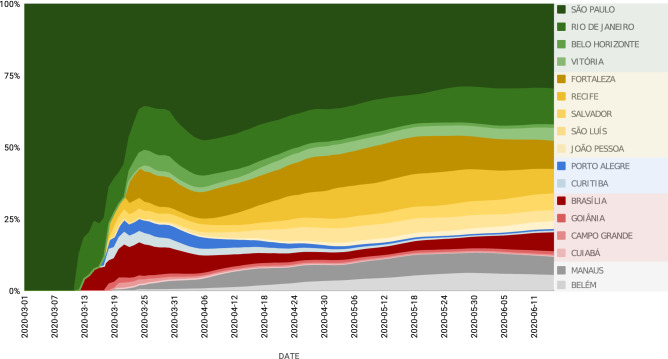
Individual contribution of the 17 state capital cities responsible for 98% of spreading of COVID-19 cases for the 5570 Brazilian municipalities, from March 1st to June 11th. Notice how São Paulo contributed to more than 80% of all cases spreading during the first weeks of March. Throughout the period until June 11th, São Paulo’s contribution never decreased below 30%. For that reason, the city was labeled as the COVID-19 super-spreader Brazilian city. Notice also the high contribution of Rio de Janeiro, Brasilia, and five state capitals in the Northeast region: Fortaleza, Recife, Salvador, São Luís, and João Pessoa. Manaus and Belém were the largest spreading cities in the North (Amazon) region, and Porto Alegre and Curitiba the most important in the South region. During this period, the contributions of Goiânia, Campo Grande, and Cuibá in the Central-West region were the largest in their region but much smaller when compared to other regions and their spreaders.

Next, we focused on identifying the major Brazilian cities contributing to the COVID-19 case spread through the Brazilian highway grid. A spatially coupled dynamical model, using human mobility data for the whole country (see “Materials and methods”), revealed that, during the first 3 weeks of the epidemic (from the last week of February to mid-March), by itself, the city of São Paulo, which is situated near the largest Brazilian international airport and is responsible for the highest highway traffic flow in the country, accounted for the spread of more than 85% of the original cases that found their way throughout Brazil (Fig. 2). Because of such a staggering initial contribution, and the fact that it never dropped below 30% during the next 3 months, São Paulo was the main Brazilian superspreader city of the SARS-CoV-2 epidemic.

Following this initial 3-week epidemic period, other major Brazilian capitals began to contribute their share to the spread of COVID-19 cases throughout the country. Thus, during the next months, cities like Rio de Janeiro (SE region), Belo Horizonte (SE), Fortaleza (NE), Recife (NE), São Luís (NE), João Pessoa (NE), Porto Alegre (S), Curitiba (S), Brasilia (CO), and Manaus (NO) all made significant contributions. By considering only the top 17 spreading cities and the highways highlighted above, we were able to account for the spread of about 98–99% of the COVID-19 cases reported in Brazil during the first 3 months of the epidemic. Since our model is based on mobile geolocation data, not road traffic, other routes were also accounted for in our study, such as the state of Amazon's widespread river-based transportation system.

Although the distributions of COVID-19 cases and deaths were significantly correlated in the initial 6 months of the pandemic (r = 0.886, p < 0.0001), our correlation analysis revealed the existence of an unaccounted residual. This meant that the distribution of deaths (Fig. 1D) could not be explained solely by the origin of the cases (i.e., the city in which the person was originally infected). Instead, to account for this residual, we had to bring to the foreground what soon became another fundamental factor in the Brazilian COVID-19 epidemic: the geographic distribution of the approximate 20 thousand intensive care unit (ICU) beds dedicated only for COVID-19 care across the entire country since February 2020.

**Figure 3:**
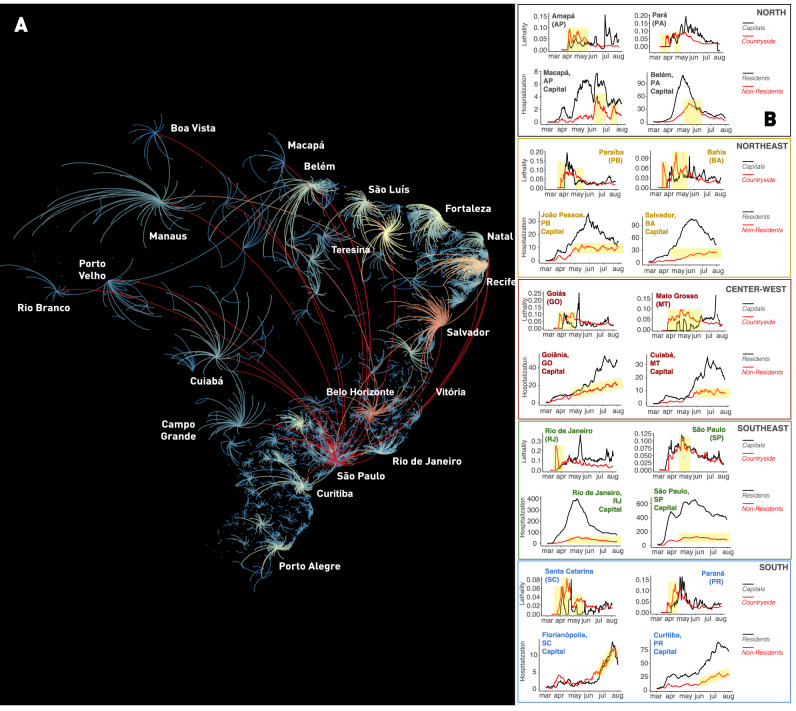
Quantification of the Brazilian “boomerang effect”. (**A**) Representation of all “boomerangs” that occurred around major Brazilian state capitals (see labels for names) and mid-size cities across the whole country. In this map, arcs represent the flow of people from the interior towards the capital. The arc color code represents the number of interior cities that sent severely ill patients to be admitted in hospitals in a capital or mid-size town; red being the highest number of locations, orange and yellow next, while a smaller number of locations are represented in light blue. Most of the flow of people represented in this graph took place through highways. Red arcs likely represent long-distance flow by airplanes. In the Amazon, most of the flow of people towards Manaus occurred by boats through the Amazon river and its tributaries. Notice that again São Paulo appears as the city with the highest boomerang effect, followed by Belo Horizonte, Recife, Salvador, Fortaleza, and Teresina. (**B**) Lethality and hospitalization data, divided for capital and interior (for lethality) and capital resident and non-resident (hospitalization), for a sample of state capitals in all five regions of Brazil. Yellow shading in the lethality graphs represent periods in which more deaths occurred in the interior, in relation to the capital. In the hospitalization graphs, yellow shading depicts periods of increasing admission of people residing in the countryside to the capital hospital system. The overall flow of people from capital to the interior and back to the capital characterized the boomerang effect, targeting the hospital system of the capital city. Notice that the boomerang effect was pervasive all over the country, occurring in every Brazilian state. Map generated using Gephi (https://gephi.org/, see Bastian et al.^48^).

In Brazil, the vast majority of tertiary hospitals, and hence the largest share of intensive care unit beds, is located in state capitals, their metropolitan areas, and a handful of mid-sized towns in each state's interior. By tracking the flow of COVID-19 cases since the beginning of April, and taking into account patient admissions in ICUs nationwide, we were able to identify, in mid-June, a very peculiar flow of people all over Brazil (Fig. 3A). As mentioned above, during the initial stages of the epidemic, COVID-19 cases began to grow rapidly in the state capitals where major international airports were located. As smaller towns are highly dependent on state capitals for accessing public services, including health care, and for the acquisition of goods and services, and as cases increased in the capitals, many infected people moved towards the vast Brazilian interior through the highway grid. As a consequence of the interiorization of COVID-19 cases, the flow of severely ill patients from the countryside to capitals took place all over Brazil (Fig. 3A,B), multiple times during the first 6 months of the pandemic. We named the overall phenomenon that created the flow of infected people from state capitals to the interior and then brought severely ill patients back to the state capitals and large Brazilian cities, “the boomerang effect”.

Figure 3A summarizes all major boomerangs that took place throughout Brazil during the past 6 months. Arcs represent the countryside origins of the most intense patient flows towards state capitals and mid-sized interior cities for the entire country. Once again, São Paulo appears as the city with the highest boomerang effect, followed by Belo Horizonte, Recife, Salvador, Fortaleza, and Teresina, all the latter being state capital cities (Fig. 3A). Boomerangs were so pervasive throughout the country that they triggered significant surges in hospital admissions in most state capitals in all Brazilian regions (see yellow highlights in Fig. 3B), leading to lethality peaks in each of these cities (Fig. 3B). Moreover, the boomerang flow was not restricted to roads and highways. For instance, in the Amazon rain forest, severely ill people were transported by boats of all sorts, via its large rivers, from many small riverside communities, towards the two largest Amazon cities, Manaus and Belém (see Fig. 3A).

Severe Acute Respiratory Infections (SARI) data revealed that São Paulo was the city with the highest influx of non-resident patients and received patients from 464 different cities all over Brazil, followed by Belo Horizonte (351 cities), Salvador (332 cities), Goiânia (258 cities), Recife (255 cities), and Teresina (225 cities). São Paulo was also the city that sent more residents to be hospitalized in other cities (158 cities), followed by Rio de Janeiro (73 cities), Guarulhos (41 cities), Curitiba (40 cities), Campinas (39 cities), Belém (38 cities), and Brasília (35 cities). As predicted, the influx of patients received by a city was significantly correlated with the number of ICU beds available (r = 0.41, p < 0.001, both normalized by population size). The accumulated number of deaths was positively correlated to both the number of influx connections (r = 0.65, p < 0.001; log-transformed data) and the number of outflux connections (r = 0.64, p < 0.001; log-transformed data). This indicates that cities that were highly connected to the health system network, either by receiving from or sending patients to other cities, also experienced a higher number of COVID-19 deaths.

**Figure 4:**
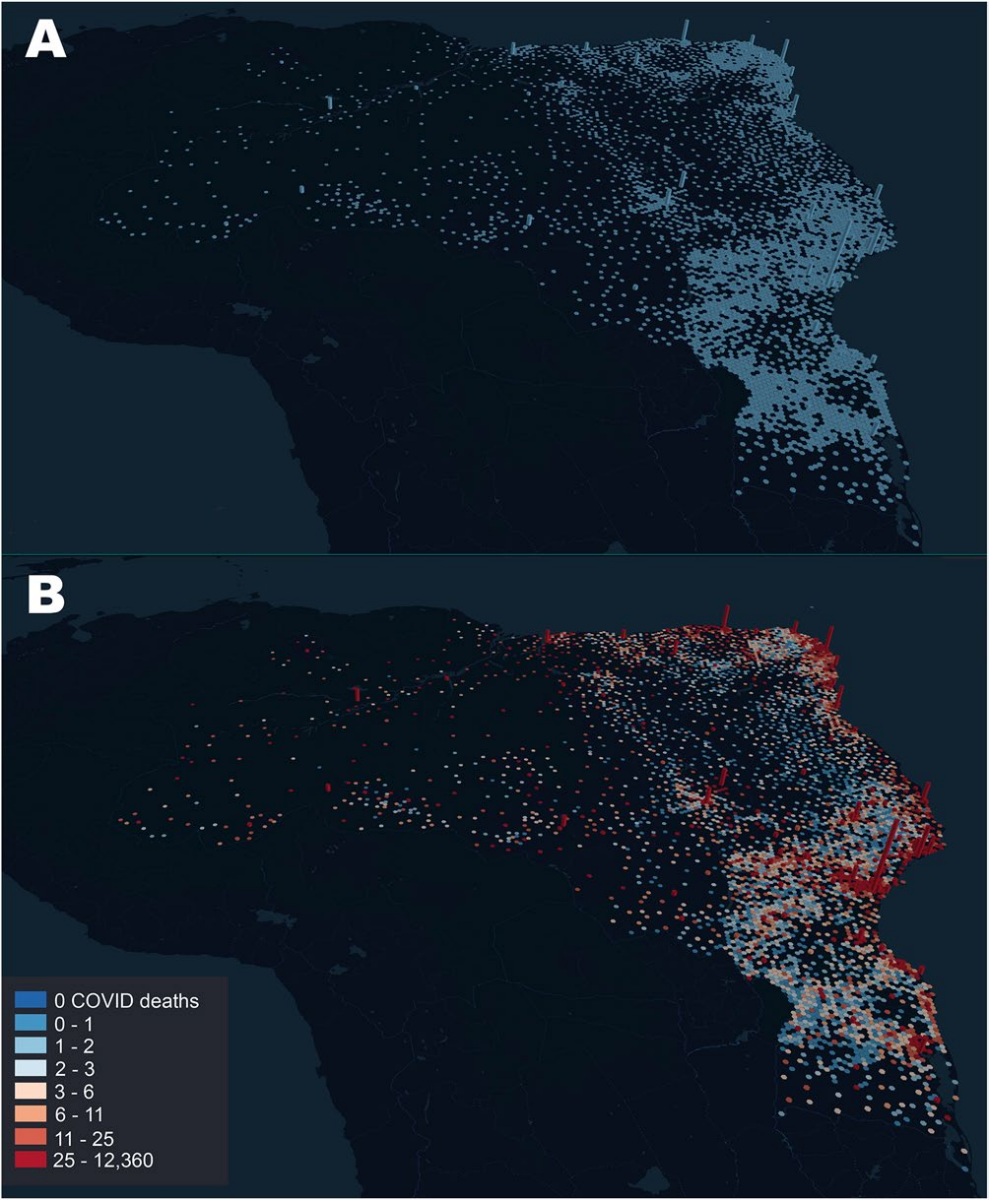
(**A**) Distribution of ICU beds across all Brazil. Bar height is proportional to the number of ICU beds in each city. Notice how the coastal state capitals accumulate most of the ICU beds in the whole country, with much fewer beds available in the interior of most states. The city of São Paulo exhibits the larger number of ICU beds in the whole country. (**B**) Superimposition of the COVID-19 death distribution (color code legend on the left lower corner) on top of the ICU bed distribution as seen in (**A**). For each bar, its height represents the number of ICU beds in a city, while color represents the number of deaths that occurred in that city. Again, the city of São Paulo, which has by far the highest number of ICU beds, accumulated the highest number of COVID-19 related fatalities, followed by state capitals like Rio de Janeiro, Fortaleza, Brasilia, Salvador, Manaus, Recife, and Belém. Maps generated using the online resources available at http://kepler.gl/.

At this point, we decided to test whether the skewed geographic distribution of ICU beds across the country could account for the death distribution residual we described before. Figure 4A illustrates the spatial distribution of ICU beds across all of Brazil. Once this distribution was plotted on top of the COVID-19 death distribution (Fig. 4B), we observed that the two variables were highly correlated (r = 0.9255, p < 0.0001). In other words, independently of their original residence, either interior towns or large cities, a significant number of people died in the state capitals and mid-size cities where tertiary hospital facilities and ICU beds were highly concentrated. Therefore, as a result of the boomerang effect, a significant number of severely ill patients had to migrate to larger cities and, eventually, a high fraction of them perished there. Combined with the deaths of the residents of large cities, the widespread boomerang effects contributed decisively to the geographic skewing of the COVID-19 death distribution in all of Brazil.

A multi-linear model that considered both COVID-19 cases and ICU beds as dependent variables to explain deaths showed that, while both variables contributed significantly to explaining COVID-19 lethality, on average, each ICU bed accounted for 1.23 deaths (95% CI = [1.139, 1.333]) by July 1st, and 3.8 deaths (95% CI = [3.685, 3.922]) when the period of analysis was extended to September 12th (see Supplementary Tables 3 and 4 for details).

## Discussion

Overall, we identified three major factors that concurrently accounted for most of the early stages of the COVID-19 pandemic dynamics in Brazil. From its original entry at all major Brazilian international airports during March^2^, SARS-CoV-2 spread first to the large metropolitan areas of state capitals located next to these airports. From that point on, after community transmission was established and began to rise exponentially in these large cities, and given that no major road blocks were implemented during the early months of the epidemic, a small group of these state capitals began spreading SARS-CoV-2 to the entire country, through the extensive highway grid that covers all of Brazil. By itself, São Paulo, the city with the highest population in Brazil, emerged as the country’s superspreader city par excellence, accounting for the largest case spreading influence throughout the next 3 months. A small group of other 16 spreading cities contributed to the seeding of initial cases throughout the country via a subset of 26 major federal highways. This highway-driven spread was the primary mechanism through which initial cases arrived in all Brazilian cities. Thus, in about 30 days, SARS-CoV-2 was transported to all five regions of the country, across Brazil’s north–south axis, a distance of roughly 5313 km.

Despite the use of secondary data and Brazil's low testing levels, especially at the beginning of the pandemic, we identified clear geographical patterns that accounted for the spread of the new coronavirus cases and deaths at the countrywide scale. Although the use of secondary data provided by many distinct health authorities at the state and federal levels, along with limited testing, imposed uncertainty to our analyses due to heterogeneity in data quality and completeness, it is important to emphasize that such secondary datasets have been compiled by institutional and research networks with a broad coverage over the country (see “Materials and methods”). As such, they represent the best currently available sources of information to depict broad scale patterns of COVID-19 cases and deaths in Brazil. In this context, even though synthesizing large amounts of secondary information poses a major challenge from the methodological standpoint, it has proved to be a fundamental approach to enlighten the large-scale processes shaping epidemical dynamics in continental countries like Brazil, by providing insights into effective measures, such as strategically designed roadblocks, to mitigate the spread of eventual future pandemics.

Understanding the dispersal pathways followed by a contagious disease over large continental areas over which the flow of people is not limited by political borders, such as Brazil, the European Union or the United States, requires us to face a multi-factor complex system that imposes numerous challenges for mathematical modeling attempts. Here, we implemented a dynamical model that used human mobility, based on georeferenced mobile data, to account for the regional spreading of COVID-19 cases throughout Brazil. The same approach was used to further investigate human mobility patterns through the entire Brazilian federal highway network. Our analysis confirmed yet again the extreme relevance of human mobility in spreading infectious diseases^14,21,26,27^. While known to be relevant for regional spread, in the literature, human mobility is usually investigated on a city level, considering the concept of social distancing as a non-pharmaceutical Intervention (e.g.^3,28–31^. And even though several studies have tried to analyze the spreading of COVID-19 among cities, these attempts focused solely on accounting for the observed COVID-19 cases and deaths (see, for example, references in^32^, without identifying the actual mobility paths that explained the spreading of cases. This latter issue became even more important recently when studies demonstrated the critical relevance of interventions on such regional mobility flows in China and the USA as a possibly effective way to reduce the spreading of COVID-19 cases. For instance, Mu^17^ discussed how constraints on inter-city travel (reaching a 70% reduction in people flow) in China slowed the spread of COVID-19 cases throughout the country. Furthermore, as we did in the present work, Davis et al.^16^ employed a complex network model based on human mobility to inspect the domestic seeding of COVID-19 cases within the USA. Their analysis revealed that, even though international travel was a key driver to the initial disease spread in certain metropolitan areas, many states were infected by domestic travel flows. Our work identified 17 spreader cities in Brazil and also highlighted the main 26 roads that acted as dispersion paths for SARS-CoV-2 in Brazil, and that should have become potential targets for interventions early on in the epidemic. Such interventions could have encompassed, for example, optimized road blocks or selective traffic control of non-essential travel. Our data also corroborated, at a national level, a recent analysis of the spreaders of COVID-19 cases to the interior of the state of Pernambuco, which implicated a major transverse federal highway, BR 232, as well as other smaller state roads^20^.

Further investigation on the roles of super-spreader cities in the course of the early stages of epidemiological dynamics should take into account other features of the complex and hierarchical organization of Brazilian rural and urban territories^33^. For example, the concept of a super-spreader city can be refined to account for epidemiological hotspots encompassing several interconnected metropolitan territories that are common in several states, such as São Paulo, Rio de Janeiro and Minas Gerais. Indeed, the contiguous diffusion among highly interconnected cities within metropolitan areas has been shown to act as a complementary spreading mechanism shaping geographical patterns of COVID-19 cases along with the hierarchical propagation via long-distance trips involving cities of regional relevance^34^.

Since Brazil’s air space remained open for international (and national) travel until the end of March and no travel restrictions were imposed on the main roads leaving the superspreading city (São Paulo) and other major Brazilian state capitals, Brazilian highways provided transportation for people infected by the new coronavirus to all parts of the country for a full month after the first case was reported in São Paulo (February 26th). Thus, by the time (mid- to late-March) state governments began issuing decrees imposing social isolation measures for all people (except for those deemed essential workers), all the pre-conditions for COVID-19 community transmission around the entire country were already in place. Our analysis revealed that traffic through federal highways alone contributed to 30% of this COVID-19 case spread. However, since we did not analyze state and municipal roads like other studies^20^, the contribution of roads to the movement and spread of infected people all over Brazil is likely to be much higher.

Considering these results, one can conclude that had the Brazilian federal government decided to decree an early national lockdown in March 2020, including the establishment of road blocks for testing and detecting infected incoming travelers, as well as restrictions to non-essential traffic (e.g., inter-municipal and inter-state buses and private passenger cars) on major Brazilian federal highways, the number of COVID-19 cases and deaths would be significantly lower throughout the country. By the same token, if São Paulo’s government had decreed a complete lockdown of the city (and the state of São Paulo) and imposed severe restrictions to non-essential traffic through the main highways that originate or cross the city and its metropolitan region (in early March 2020), the number of COVID-19 cases and deaths during the early phases of the Brazilian pandemic would be much lower, throughout the country. In summary, had all these preventive non-pharmacological actions taken place in early March, Brazil would almost certainly have been spared the largest humanitarian tragedy of its history.

As community transmission began to happen in earnest, and case numbers rose rapidly in the countryside towns, a growing number of severely ill patients began to overwhelm smaller local hospitals that lacked enough qualified personnel and ICU beds to manage such an unusually high demand for critical care. Under these dire circumstances, a significant portion of these patients had to be transported to the large state capitals in search of better specialized care and available ICU beds. In some states, like São Paulo, this patient migration was also directed to mid-sized towns with large public university hospitals, such as Ribeirão Preto and Campinas. We also observed that the distribution of COVID-19 related deaths overlapped quite well with the equivalent spatial distribution of ICU beds throughout Brazil. Patient flow related to the medical care system in Brazil is well-known, particularly regarding the widespread need of long-distance travel for high complexity care^35^, which has the potential to disseminate and select resistant pathogen strains^36^. This structure affected the COVID-19 spreading pattern such that the higher the number of ICU beds in a city, the higher the number of deaths recorded from March to September 2020. A nationwide analysis of the cause of this overlap, which we named the boomerang effect, revealed that while state capitals, mostly located on the country’s Atlantic coast, provided the primary sources of infections to mid- and small-sized towns situated in Brazil’s vast interior,. As a result of this gigantic human flow, people from interior towns began to account for a large percentage of patient admissions in both public and private hospitals in state capitals. Thereafter, several of these hospitals in both mid-sized towns and state capitals became overwhelmed. Indeed, the health systems of some cities subject to boomerang effects, like Manaus^37,38^, where the boomerang effect was mainly operating through the Amazon River, collapsed altogether. During a few weeks, ICU bed occupancy reached more than 90% in multiple Brazilian state capitals, an event that has never been seen before in Brazilian medical history^39^. Although most state governments tried to mitigate this crisis by quickly adding new infirmary and ICU beds to their hospitals, the lack of specialized personnel, individual protection, and sophisticated medical equipment, such as modern artificial ventilators, reduced the efficiency of such countermeasures. As a result, each ICU bed available in the country for COVID-19 accounted, on average, for 1.23 deaths by July 1st and for 3.8 deaths by September 12th, according to our partial correlation analysis. An independent investigation, reported in a technical note, also identified the boomerang effect herein reported.

The Brazilian federal health care system, known as the “Sistema Único de Saúde” (SUS; in English: Unified Health System) was created more than 30 years ago^40^ with the mission of ensuring the constitutional right of free health care to every Brazilian citizen anywhere in the country. Today, SUS constitutes the only option through which 7 out of 10 Brazilians have access to high-quality medical care for free^41^. Yet, the COVID-19 epidemic crisis exposed the inadequacy of the policy of concentrating the largest share of tertiary hospital facilities and ICU beds in a handful of mid-sized towns and state capitals throughout Brazil. Although our study did not address this issue directly, our findings suggest that had the geographic distribution of ICU beds been less skewed toward big cities, many more lives could have been saved throughout the country. Indeed, critically ill patients in less populated areas would have had regional access to ICU beds and, therefore, would have received quicker treatment and had a better chance for improved clinical outcomes. Regional access to specialized healthcare would also have reduced the demand for critical logistical resources and medical personnel necessary for transporting such seriously ill patients over long distances to metropolitan hospitals, eliminating the widespread boomerang effect documented here.

In conclusion, our work identified the key factors that allowed the rapid spread of the SARS-CoV-2 across Brazil during the early stages of the ongoing pandemic. It also identified candidate processes that are likely to play key roles in shaping the geographical patterns of cases and deaths, not only for COVID-19, but also for any contagious disease spreading over a country in which: (1) the logistics and passenger transportation are highly dependent on a highway system; and (2) the responses for public health emergencies rely mostly on the structure of a public health system. Our results sustain the notion that a restricted number of “super-spreader cities'', key federal highways and the extremely biased spatial concentration of ICU bed availability, seeded the pattern of spatial and temporal spread of the pandemic over the Brazilian countryside. In the years to come, this notion should be considered a departure point for the design of urgently needed public policies aimed at preparing Brazilian federal and state-level health institutions to coordinate robust non-pharmacological responses when facing severe nationwide epidemiological emergencies, such as the ongoing SARS-CoV-2 pandemic. Such responses could include, for example: (1) the planning and implementation of strategically placed roadblocks and extensive border closures between states as soon as epidemiological emergencies are officially declared; (2) funding for the development of information systems and associated research networks to ensure the availability of high-quality datasets to inform the design case-specific mitigation strategies at the federal, state and regional levels; and (3) the gradual decentralization of the availability of ICU beds and associated services to cover a larger territory of the Brazilian countryside that is currently unassisted in terms of complex medical care, hence making the access to public health truly universal—a central milestone of the 1988 Brazilian Constitution.

## Materials and methods

### Data sources for COVID-19 cases, deaths and hospitalization

We obtained data describing the temporal evolution of COVID-19 cases and deaths in Brazil at the municipal and state levels from several sources, including the Brazilian Ministry of Health^42^, official daily epidemiological bulletins issued by each Brazilian state^43^, and other sources as compiled by Cota et al.^44^. Both cases and death data refer to notifications per day. To compute incidence (cases per 100,000 inhabitants), we used population size estimates for each of the 5570 Brazilian municipalities for 2019. Such population size estimates were aggregated to allow the computation of COVID-19 at the state level. The data regarding COVID-19 reported cases and deaths included the period from 25/02/2020 until 12/09/2020, with sub-periods mentioned, when appropriate, in the main text. ICU bed data took into account both adult and pediatric beds that were used specifically for COVID-19 patients. This data is available at DATASUS CNES (Physical Resources, Hospital Complementary Beds). We used the information for July 2020, which was the last period available.

We also employed the Ministry of Health’s data on Severe Acute Respiratory Infections (SARI) data, in which COVID-19 cases represents close to 98% of the data in 2020, to obtain detailed information about patients’ residence and hospitalization location throughout Brazil (https://opendatasus.saude.gov.br/dataset/bd-srag-2020). The SARI data contains only a subset of the official reported COVID-19 cases since they cover only hospitalized cases. The weighted directed network of patient flux was built with SARI data. Because the weight of the influx (i.e., in-degree) and outflux (i.e., out-degree) showed high positive correlations with population sizes, we used the unweighted network to explore the effects of city interaction patterns on COVID-19 deaths.

All correlation analyses were performed with Pearson correlation (r) on original or transformed variables (normalized by population or log-transformed). A multi-linear model considering deaths as dependent on COVID-19 cases and bed availability was also considered (see Supplementary Tables 3 and 4). While some multicollinearity was observed, the condition number was deemed small enough to allow the interpretation of the results.

### Data source for the Brazilian federal road system

The shapefile with geospatial data describing the Brazilian federal roads' distribution was obtained from the Brazilian National Road System. Roads were categorized according to the official typology: longitudinal roads (codes starting with 1, as in BR101) are those crossing the country from north to south; transversal roads which cross the country from east to west (codes starting with 2); diagonal roads (codes starting with 3); connector roads are shorter roads connecting major federal roads (codes starting with 4); and radial roads, those departing from Brazil’s capital, Brasilia, which has a central geographical position (codes starting with 0).

### Highway multi-linear model

To investigate the most representative highways concerning the COVID-19 spatial distribution pattern, we built a multi-linear model on a city level. Highways were included in the model as dummy variables, considering 1 for cities it crosses, and 0 for cities it does not cross. Model selection started from all federal highways, and then a 3 step filtering process was performed. The first filter eliminated variables (representing highways) with coefficients with statistical p-values larger than 0.10, then two subsequent steps of elimination were performed for variables with p-values larger than 0.05. The resulting multi-linear model significantly adjusted 26 highways (R2 = 0.3, p < 0.025 for all variables) when the response variable was the accumulated COVID-19 cases on September 12th, 2020, and significantly adjusted 16 highways (R2 = 0.23, p < 0.015 for all variables) for accumulated COVID-19 deaths on the same date. Model details in Supplementary Tables 1 and 2.

### Spatial spreading model

The spatial spreading of COVID-19 throughout the country was modeled following the approach described in Peixoto et al.^21^. This approach is based on a complex mobility network of all Brazilian cities coupled with a compartmental model containing infected and susceptible individuals (SI model), adequate for simulations of initial epidemic dynamics. The mobility data is based on individual pairwise mobile geolocation data, resulting in multiple daily travel information between cities, collected from the Brazilian company Inloco^45^. The compartmental model consists in a simulation of a local SI model (with infection rate *r*) with added in/out flows of infected individuals to cities connected via the mobility data. The mobility data, with methodological aspects described in details in Peixoto et al.^21^, describes counts of trips between Brazilian cities, summing tens of millions daily trip counts between all major Brazilian cities. The main difference between this study and Peixoto et al.^21^ is that here we consider all Brazilian cities, whereas Peixoto et al.^21^ only investigated dissemination within cities of São Paulo and Rio de Janeiro states.

We adopted an infection rate of r = 0.2 individuals per day because it provides more realistic forecasts for the initial growth of the pandemic in Brazil, compared to the initial infection rates obtained for the country (e.g.^46,47^). The flux intensity parameter proposed in Peixoto et al.^21^ was set to s = 1, that is, no compensation of the flux intensity was performed, and the real daily sampled movement counts were used to infer the mobility between cities. The code and mobility data are available at the GitHub repository https://github.com/pedrospeixoto/mdyn.

### Model of the super-spreaders

For each state capital of Brazil, a separate simulation was performed using the spatial spreading model, considering as initial condition one infected individual in the city. As discussed in Peixoto et al.^21^, the simulation encompasses the potential spreading due to mobility between cities considering a fixed starting point (one city). The simulations were run, for each capital city, from 2020-03-01 until 2020-05-01, with a result, on the final day, consisting of the potential spatial spreading pattern for each capital city. Therefore, for each capital city, we have a vector (${\overrightarrow{v}}_{j})$ indicating the potential spreading intensity with respect to all Brazilian cities. It is important to emphasize that here we used the actual observed mobility patterns of the period covered in this study, therefore our data captured lockdowns or other mobility restriction measures implemented during this period. The super-spreaders linear model was built by projecting, in the least-squares sense, the daily observed COVID-19 cases into the sub-space generated by the linear combination of the spreading patterns (vectors) obtained for each capital city. The final model, for each city (*i*) can be represented as

$$\text{I}\left(\text{t}\right)=\sum\limits_{\text{j}}{{\upalpha }}_{\text{j}}\left(\text{t}\right)\overrightarrow{{\text{v}}_{\text{j}}}$$

where $\text{I}\left(\text{t}\right)$ is the observed number of infected individuals for all locations (Brazilian cities) and ${\overrightarrow{v}}_{j}$ is a capital city spreading vector (obtained from the spatial spreading model), with dimension given by the total number of locations (Brazilian cities) and *j* varying for all capital cities. Time is considered on a daily basis, and ${{\upalpha }}_{\text{j}}\left(\text{t}\right)$ is calculated with Least Squares Approximation for each day.

As a result, we have a linear model for each day of observed COVID-19 cases, with coefficients $({{\upalpha }}_{\text{j}}\left(\text{t}\right)$) representing the degree of participation of a given city in the observed spatial pattern of COVID-19 cases. Based on the most representative 17 capital spreading patterns (largest coefficients), the super-spreaders linear model accounted for at least an adjusted coefficient of determination of 0.94 in all dates analyzed in this study (over 0.98 in the first 2 months analyzed).

### Methodological limitations

While fully coupled in space by mobility, the dynamical model adopts a simplified compartmental dynamic, with susceptible-infected only. This limits the model's ability to foresee longer periods in time compared with the many existing variants of SEIR models. However, this approach reduces the complexity in the parameter calculations and, most importantly, in the estimates of initial conditions for unobserved compartments. To compensate for this limitation, we only used the model for short periods and focused our conclusions on the spatial distribution patterns of the forecasted results rather than on the precise case count calculated.

### Dataset information and limitations

The mobile mobility dataset was provided by Inloco^45^, available upon request, and samples approximately one-fifth of the Brazilian population. While having extensive coverage of the Brazilian population, our data may have uneven distribution in space, age, and social classes. However, although adults and large cities dominate the data, samples comprise more than 90% Brazilian municipalities. To the best of our knowledge, this dataset is the largest database of the kind available for research in Brazil.

## Supplementary Information

Supplementary Information.
