# Supplementary material for: The impact of super-spreader cities, highways, and intensive care availability in the early stages of the COVID-19 epidemic in Brazil
Source: Sci Rep. 2021 Jun 21;11:13001. doi: 10.1038/s41598-021-92263-3 (PMC8217556; doi:10.1038/s41598-021-92263-3)
Supplement: Supplementary file 1 — Supplementary Information. [file 41598_2021_92263_MOESM1_ESM.docx]

**Supplementary Materials**

**The Impact of Super-Spreader Cities, Highways, and Intensive Care Availability in the Early Stages of the**

**COVID-19 Epidemic in Brazil**

Miguel A.L. Nicolelis^1-6*^, Rafael L. G. Raimundo^7^, Pedro S. Peixoto^8^, Cecilia S. Andreazzi^9^

^1^Department of Neurobiology, Duke University Medical Center, Durham, NC, USA

^2^Department of Biomedical Engineering, Duke University, Durham, NC, USA ^3^Department of Neurology, Duke University, Durham, NC, USA

^4^Department of Neurosurgery, Duke University, Durham, NC, USA

^5^Department of Psychology and Neuroscience, Duke University, Durham, NC, USA

^6^Edmond and Lily Safra International Institute of Neurosciences, Natal, Brazil

^7^Department of Engineering and Environment and Postgraduate Program in Ecology and Environmental Monitoring (PPGEMA), Center for Applied Science and Education, Universidade Federal da Paraíba - Campus IV, Rio Tinto, Paraíba, Brazil

^8^Department of Applied Mathematics, Institute of Mathematics and Statistics, University of São Paulo, São Paulo, Brazil

^9^Laboratory of Biology and Parasitology of Wild Reservoir Mammals, IOC, Oswaldo Cruz Foundation, Rio de Janeiro, Brazil

| OLS Regression Results  =====================================================================================  Dep. Variable: COVID-19 cases R-squared (uncentered): 0.300  Model: OLS Adj. R-squared (uncentered): 0.296  Method: Least Squares F-statistic: 91.17  Date: Fri, 18 Sep 2020 Prob (F-statistic): 0.00  Time: 22:34:03 Log-Likelihood: -54495.  No. Observations: 5570 AIC: 1.090e+05  Df Residuals: 5544 BIC: 1.092e+05  Df Model: 26  Covariance Type: nonrobust  ==============================================================================  Highways coef std err t P>\|t\| [0.025 0.975]  ------------------------------------------------------------------------------  10 1376.1596 602.883 2.283 0.022 194.273 2558.046  101 1923.1095 273.813 7.023 0.000 1386.329 2459.890  116 2482.4830 300.351 8.265 0.000 1893.678 3071.288  153 851.7395 353.438 2.410 0.016 158.863 1544.616  156 3553.5455 1297.440 2.739 0.006 1010.054 6097.037  20 1324.3592 598.019 2.215 0.027 152.007 2496.712  222 2092.1731 624.049 3.353 0.001 868.793 3315.553  226 1174.1774 530.969 2.211 0.027 133.270 2215.085  232 1865.1459 832.103 2.241 0.025 233.898 3496.394  272 4463.0694 578.873 7.710 0.000 3328.252 5597.887  316 1180.2546 430.952 2.739 0.006 335.419 2025.090  319 6613.6954 1317.867 5.018 0.000 4030.160 9197.231  324 2687.9860 689.525 3.898 0.000 1336.248 4039.725  364 1201.8737 457.288 2.628 0.009 305.410 2098.337  374 6591.6562 668.518 9.860 0.000 5281.098 7902.214  381 5607.5653 542.971 10.328 0.000 4543.129 6672.002  40 2631.0293 676.988 3.886 0.000 1303.868 3958.191  401 9020.0000 2151.562 4.192 0.000 4802.096 1.32e+04  408 2742.4245 966.136 2.839 0.005 848.418 4636.431  425 8005.4770 2526.973 3.168 0.002 3051.619 1.3e+04  447 1.193e+04 3055.063 3.905 0.000 5939.770 1.79e+04  448 4900.2670 2172.424 2.256 0.024 641.464 9159.070  450 1.359e+05 4535.776 29.964 0.000 1.27e+05 1.45e+05  465 2.986e+04 2513.815 11.878 0.000 2.49e+04 3.48e+04  50 9478.0408 644.509 14.706 0.000 8214.552 1.07e+04  60 2599.5477 728.586 3.568 0.000 1171.233 4027.863  ==============================================================================  Omnibus: 15534.421 Durbin-Watson: 2.002  Prob(Omnibus): 0.000 Jarque-Bera (JB): 848528010.990  Skew: 35.457 Prob(JB): 0.00  Kurtosis: 1913.787 Cond. No. 16.7  ============================================================================== |
| --- |

**Supplementary Table 1.** Output of multivariate linear model using federal highways as independent variables, defined at city level with 1 where it crosses and 0 otherwise, and COVID-19 accumulated cases up to September 12th as dependent variable. Software used: Python/stamodels.

| OLS Regression Results  =======================================================================================  Dep. Variable: COVID-19 deaths R-squared (uncentered): 0.230  Model: OLS Adj. R-squared (uncentered): 0.228  Method: Least Squares F-statistic: 103.6  Date: Fri, 18 Sep 2020 Prob (F-statistic): 7.02e-300  Time: 23:20:41 Log-Likelihood: -37659.  No. Observations: 5570 AIC: 7.535e+04  Df Residuals: 5554 BIC: 7.546e+04  Df Model: 16  Covariance Type: nonrobust  ==============================================================================  coef std err t P>\|t\| [0.025 0.975]  ------------------------------------------------------------------------------  101 81.3736 13.311 6.113 0.000 55.278 107.469  116 113.3821 14.461 7.841 0.000 85.033 141.731  20 88.1148 29.047 3.033 0.002 31.171 145.059  222 108.6331 30.229 3.594 0.000 49.372 167.895  232 127.5577 40.435 3.155 0.002 48.289 206.826  272 196.7080 28.137 6.991 0.000 141.548 251.868  308 112.3929 39.546 2.842 0.004 34.867 189.919  319 296.6364 63.094 4.702 0.000 172.948 420.325  374 289.4084 32.502 8.904 0.000 225.692 353.124  381 229.0207 26.404 8.674 0.000 177.258 280.783  40 192.6486 32.922 5.852 0.000 128.109 257.188  408 175.9069 46.982 3.744 0.000 83.803 268.011  447 376.1264 148.566 2.532 0.011 84.879 667.374  450 1986.4747 215.770 9.206 0.000 1563.481 2409.468  465 3341.9438 122.228 27.342 0.000 3102.328 3581.559  50 362.7620 31.338 11.576 0.000 301.327 424.197  ==============================================================================  Omnibus: 14643.501 Durbin-Watson: 1.999  Prob(Omnibus): 0.000 Jarque-Bera (JB): 508907991.744  Skew: 30.421 Prob(JB): 0.00  Kurtosis: 1482.554 Cond. No. 16.3  ============================================================================== |
| --- |
|  |

**Supplementary Table 2.** Output of multivariate linear model using federal highways as independent variables, defined at city level with 1 where it crosses and 0 where not, and COVID-19 accumulated deaths up to September 12th as dependent variable. Software used: Python/stamodels.

| OLS Regression Results  ==============================================================================  Dep. Variable: COVID-19 deaths R-squared: 0.846  Model: OLS Adj. R-squared: 0.846  Method: Least Squares F-statistic: 1.524e+04  Date: Fri, 18 Sep 2020 Prob (F-statistic): 0.00  Time: 23:24:26 Log-Likelihood: -30603.  No. Observations: 5570 AIC: 6.121e+04  Df Residuals: 5567 BIC: 6.123e+04  Df Model: 2  Covariance Type: nonrobust  ==============================================================================  coef std err t P>\|t\| [0.025 0.975]  ------------------------------------------------------------------------------  const -3.6450 0.795 -4.586 0.000 -5.203 -2.087  beds 1.2363 0.049 24.991 0.000 1.139 1.333  cases 0.0391 0.001 44.475 0.000 0.037 0.041  ==============================================================================  Omnibus: 13139.500 Durbin-Watson: 1.869  Prob(Omnibus): 0.000 Jarque-Bera (JB): 525256895.055  Skew: 22.747 Prob(JB): 0.00  Kurtosis: 1506.714 Cond. No. 2.33e+03  ============================================================================== |
| --- |

**Supplementary Table 3.** Output of multivariate linear model using hospital beds and accumulated COVID-19 cases (until July 1st, 2020) as independent variables, and COVID-19 accumulated deaths as dependent variable. Software used: Python/stamodels.

| OLS Regression Results  ==============================================================================  Dep. Variable: COVID-19 deaths R-squared: 0.876  Model: OLS Adj. R-squared: 0.876  Method: Least Squares F-statistic: 1.960e+04  Date: Mon, 05 Oct 2020 Prob (F-statistic): 0.00  Time: 13:51:06 Log-Likelihood: -32555.  No. Observations: 5570 AIC: 6.512e+04  Df Residuals: 5567 BIC: 6.514e+04  Df Model: 2  Covariance Type: nonrobust  ==============================================================================  coef std err t P>\|t\| [0.025 0.975]  ------------------------------------------------------------------------------  const -1.0477 1.138 -0.921 0.357 -3.278 1.183  leitos 3.8033 0.060 63.042 0.000 3.685 3.922  casos 0.0142 0.000 29.167 0.000 0.013 0.015  ==============================================================================  Omnibus: 14847.266 Durbin-Watson: 1.981  Prob(Omnibus): 0.000 Jarque-Bera (JB): 874556182.692  Skew: 31.261 Prob(JB): 0.00  Kurtosis: 1943.200 Cond. No. 5.21e+03  ============================================================================== |
| --- |

**Supplementary Table 4.** Output of multivariate linear model using hospital beds and accumulated COVID-19 cases (until September 12th, 2020) as independent variables, and COVID-19 accumulated deaths as dependent variable. Software used: Python/stamodels.
